# Starch and Table Salt Sold as Neosalvarsan

**Published:** 1916-12

**Authors:** 


### Starch and Table Salt Sold as Neosalvarsan.

The recent indictment by the Federal grand jury in Newark, N. J., of "Dr." Jean F. Strandgaard, of Toronto, Canada, and George F. Hardacre, of Toronto, and a steward on the steamship "United States," has revealed to Chief Inspector E. R. Norwood, of the customs service in New York, what he believes to be a widespread conspiracy to defraud the government out of customs revenue by smuggling salvarsan and neosalvarsan into the United States.

A most serious feature of this matter is the discovery by Inspector Norwood that these men also had in their possession a large quantity of spurious neosalvarsan. Upon analysis by the government experts, the contents proved to be starch in the majority of the ampules and stained table salt in the others.

A further investigation showed that during July, 1916, Strandgaard had 15,000 ampules made in Jersey City, which upon his instructions were filled by the glass blower with either starch or salt. A remarkable coincidence is that during August and September, and as recently as the time Strandgaard was arrested in New York, physicians and drug stores all over the Middle West and the East were approached by women trying to sell, on the one pretense or another, the frauds made for Strandgaard. These spurious products were put up in imitation of either the German or particularly the English package, as marketed by the German manufacturers in England before the war, in square pasteboard cartons. They did not appear in round aluminum packages, like the American package. They are very cleverly executed, and their outside appearance even led experienced physicians to be deceived.

The product has been sold in New York, Chicago, Milwaukee, Cincinnati, Peoria, Kalamazoo, Detroit, Terre Haute and Mobile, and other Western and Southern cities, and is undoubtedly still being peddled on account of the great profits accruing to the saleswomen.

There is no need to call the attention of physicians to the dangers connected with the use of such frauds. In view of the serious and possibly fatal results which would follow the administration of these fraudulent salvarsans, it is incumbent upon medical men who have any information about the distribution or sale of these frauds to communicate with Chief Inspector E. R. Norwood, U. S. Customs House, New York, at their earliest opportunity, or, in case of emergency, with the local police authorities.

